# Classification of brain tumor types through MRIs using parallel CNNs and firefly optimization

**DOI:** 10.1038/s41598-024-65714-w

**Published:** 2024-07-01

**Authors:** Chen Li, Faxue Zhang, Yongjian Du, Huachao Li

**Affiliations:** 1 Department of Neurosurgery, Shandong Provincial Third Hospital, Shandong University, No.12 Wuyingshan Middle Road, Jinan, 250031 Shandong China; 2 Department of Neurosurgery, The Fifth People’s Hospital of Jinan, No.24297, Jingshi Road, Jinan, 250022 Shandong China

**Keywords:** MRI image segmentation, Classification, Firefly optimization, Brain tumor, Convolutional neural network, Cancer, Engineering, Mathematics and computing

## Abstract

Image segmentation is a critical and challenging endeavor in the field of medicine. A magnetic resonance imaging (MRI) scan is a helpful method for locating any abnormal brain tissue these days. It is a difficult undertaking for radiologists to diagnose and classify the tumor from several pictures. This work develops an intelligent method for accurately identifying brain tumors. This research investigates the identification of brain tumor types from MRI data using convolutional neural networks and optimization strategies. Two novel approaches are presented: the first is a novel segmentation technique based on firefly optimization (FFO) that assesses segmentation quality based on many parameters, and the other is a combination of two types of convolutional neural networks to categorize tumor traits and identify the kind of tumor. These upgrades are intended to raise the general efficacy of the MRI scan technique and increase identification accuracy. Using MRI scans from BBRATS2018, the testing is carried out, and the suggested approach has shown improved performance with an average accuracy of 98.6%.

## Introduction

An abnormal and cancerous cell accumulation is called a brain tumor. The human skull is a strong and vital organ that protects the brain. Any aberrant development of brain tissue within the skull's constrained borders might result in a variety of issues. There are two types of brain tumor classifications: benign and malignant^1^. The primary functions of the human anatomy, including respiration, muscular and sensory function, are controlled by the nerve cells and tissues that make up the brain^2^. Using MRI to detect and classify brain tumors is an essential part of medical therapy. Information about anatomical structure is provided by this process for treatment planning. Brain atlas building and brain modeling may benefit from tumor segmentation. Despite the availability of several technologies and their excellent results, precisely describing and segmenting abnormalities in medical imaging may be challenging because to the variability of tumor location, intensity, and form^3^.

Image segmentation plays a crucial role in therapeutic picture evaluation and analysis, focusing on object recognition and region of interest (ROI) selection. As a result, it greatly aids in monitoring, diagnosis, and therapy^4^. Different kinds of volumetric data are found using MRI segmentation. When compared to other medical images that are available, it provides precise data and is beneficial for a variety of medical image applications. Compared to other medical imaging, the contrast of an MRI provides the best results. Because an MRI represents an image in its compressed form, extracting its features is essential. Various feature extraction techniques are applied, enabling the classifiers to distinguish between normal and pathological tumors^5^. Many attempts have been made recently in studies to use tumor segmentation from medical pictures to diagnose brain tumors. In the medical field, brain tumor segmentation using machine learning is crucial for accurate disease detection. Although automated tumor segmentation has been shown to be effective, brain tumor segmentation is neither offered nor used in clinical settings. The goal of the work is to use digital image processing methods to detect tumor areas from brain MRIs. The region of the tumor is then calculated using symmetry analysis and a totally automated mechanism^6^. Medical imaging patterns may now be recognized and categorized thanks to recent advancements in machine learning, particularly deep learning. One example of this field's achievements is its capacity to collect and extract information from data instead of depending on specialized expertise or academic publications^7–9^. Because of its many layers and superior diagnostic accuracy while processing a large number of input pictures, convolutional neural networks (CNNs) are presently the most efficient image processing techniques^10,11^. Autoencoders are an unsupervised learning method that employ neural networks to speed up representation learning. Remarkably, a wide range of deep learning and machine learning algorithms have been used for cardiovascular stenosis detection as well as for the identification of malignancies, including lung tumors. Performance assessments have also shown to be quite accurate in diagnosing problems^12–15^.

In this paper, an attempt has been made to identify the type of brain tumors from MRI scans using a combination of optimization and an ensemble of convolutional neural networks. However, there is still room for improvement with the present approaches. Two innovations that can be used to increase identification accuracy have been described in this research. The first innovation consists of the introduction of a novel segmentation technique that employs the FFO algorithm and multiple criteria to determine segmentation quality of images. In order to classify the features of tumor areas and determine the type of tumor, the second innovation combines two different forms of convolutional neural networks. When employing this mix of models instead of each one independently, the accuracy is increased. Below is a summary of our main contributions.

- Combination of convolutional neural network and optimization techniques for tumor identification.
- Introduction of a novel segmentation technique using the FFO algorithm.
- Combination of two different forms of convolutional neural networks for tumor classification.

The paper proceeds as follows: Section "Literature survey" looks at similar works. In Section "Research methodology", the suggested method is described; in Section "Implementation results", the outcomes of its execution are given; and in Section "Conclusion", conclusions are reached.

## Literature survey

Using a dataset of 3064 MRI images of 233 individuals with brain tumors, Phaye et al.^16^ created diversified capsule networks (DCNet + +) and capsule algorithm networks (DCNet). By using a hierarchical design and a deeper convolutional network, the DCNet model increased accuracy to 95.03%, while the DCNet +  + model did the same. A CNN model that can identify meningiomas, gliomas, and pituitary tumors was created by Pashaei et al.^17^. The model had four convolutional layers, batch normalization layers, pooling layers, and fully connected layers. The model was 93.68% accurate and had a learning rate of 0.01 when compared to other methods. An automated technique for classifying brain tumor kinds in radiologists and doctors was created by Gumaei et al.^18^. With an accuracy rate of 94.23%, the research analyzed a collection of 3064 MRI pictures from 233 individuals. Nevertheless, a comparison analysis using other methods was not carried out in this research.

Multi-CNNs, a hybrid of CNN and multimodal information fusion detection techniques, were created by Li et al.^19^. This method enhanced the tumor detection process's correlation coefficient, specificity, and sensitivity. The Whale Hawks Optimization (WHHO) algorithm was presented by Rammurthy et al.^20^ as an efficient brain tumor identification method that combines the Whale Optimization (WOA) and Harris Hawks Optimization (HHO) methods. For tumor area segmentation on MRI images, it incorporates statistical and textural information together with pixel data from the images using cellular automata and rough set theory. Even with low-quality photos, this technique may enhance image quality.

A deep CNN model was used by Haq et al.^21^ to suggest a deep learning-based diagnostic technique for the categorization of brain tumors, namely meningiomas, gliomas, and pituitary tumors. To improve predictive capacity, the approach combines data augmentation and transfer learning strategies. With a 99.90% accuracy rate, the ResNet-CNN model may be used in Internet of Things healthcare for brain tumor identification and classification. DensNet201 and Inception-v3, pre-trained deep learning models, were created by Noreen et al.^22^ to aid in the early diagnosis of brain cancers. Concatenating features from the Inception-v3 model, using the softmax classifier to identify tumors, and extracting pre-trained DensNet201 from many blocks are the steps involved in the process.

Despite employing fewer medical pictures, Hashemzehi et al.^23^ were able to achieve great classification performance by training a hybrid CNNNADE model with 3064T1-weighted contrast-improved images of three different forms of brain tumors. With pre-trained SqueezeNet architecture for feature extraction and ELM classification, Özyurt et al.^24^ proposed the SR-FCM-CNN approach for brain tumor identification, attaining 98.33% accuracy, a 10% improvement over segmenting brain tumors using FCM without SR. BrainMRNet, a CNN technique made up of attention modules and a residual network, was first presented by Toğaçar et al.^25^. Preprocessing, feature extraction, and picture augmentation were done. When compared to VGG-16, GoogleNet, and AlexNet models, the model achieved a 96.05% classification accuracy in the effective detection of brain cancers using MR images. AlexNet, GoogLeNet, and VGGNet are the three CNNs that Rehman et al.^26^ looked at. Using a linear classifier, automatic characteristics were categorized in the last stage. Utilizing data augmentation methods, the sample size was increased and the risk of over-fitting was reduced. Based on the assessment data, the VGG16 approach has the highest accuracy (98.69%) when compared to other methods.

A CNN with 22 layers was used by Badža et al.^27^ to categorize pituitary tumors, meningiomas, and gliomas. Thirty-six T1-weighted contrast-enhanced MRI pictures made up the imaging dataset. For the purpose of classifying and segmenting medical images, Narmatha et al.^28^ created a fuzzy brain storm optimization algorithm that prioritizes cluster centers based on their importance for the best outcomes. Noreen et al.^22^ developed a multi-level feature extraction and concatenation technique for early brain tumor diagnosis utilizing trained deep learning models, Inception-v3 and DensNet201. This approach addresses problems like as big datasets and low-quality medical pictures by increasing model capabilities while reducing processing complexity. A secure CNN model was reported by Mohammad et al.^29^ to predict brain cancers from MRI data. Blockchain layers and characteristics retrieved from the scans are used in the model. With a prediction accuracy of 99.75%, precision of 97.94%, and recall of 98.73%, the model achieves the best accuracy. Future developments in the model's recognition performance might include sophisticated LLB-CNN integration and hashing techniques, while its accuracy and consistency stay intact. An overview of the studies on brain tumor detection is provided in Table 1.

**Table 1:** Research on models for the identification of brain tumors.

References	Model	Features	Challenges
Phaye et al.^16^	Differentiated capsule networks (DCNet + +) and dense capsule networks (DCNet)	Outline a strategy for enhancing results with a densely layered network	To improve classifier execution, computational complexity needs to be decreased
Pashaei et al.^17^	KELM	Constructing an algorithm that uses the CNN and KELM to extract and classify features	Not mentioned
Gumaei et al.^18^	Regularized extreme learning machine (RELM)	Utilizing a hybrid feature extraction technique to categorize brain tumors	Absence of comparison between the machine learning technology employed in this research and alternative approaches
Li et al.^19^	CNN	Several nodes were used to extract various pieces of information	The level of computational complexity is extremely high
Rammurthy et al.^20^	WHHO-based DeepCNN	Using cellular automata and rough set theory to segment data improves results	Existing techniques necessitate a big amount of training data and have a high computational cost
		Statistical and textural features aid in the diagnosis of brain tumors	The segmentation of tumorous areas from brain MRI takes time and has an impact on accuracy
Haq et al.^21^	CNN	The proposed methodology increases brain tumor classification accuracy in IoT healthcare	Not mentioned
Noreen et al.^22^	Deep Learning	The softmax classifier was used to categorize the brain tumors	It does not use fine-tune methods on pre-trained models that have been trained with a large number of layers
		Several DensNet blocks were used to extract the features	
Hashemzehi et al.^23^	Neural autoregressive distribution estimation (NADE) and CNN	The features that are useful for categorizing an image are extracted	There aren't enough medical photos online
Özyurt et al.^24^	Fuzzy C-means and CNN	With no loss of performance, it can make decisions quickly	It can't be used as a classifier in a number of instances
Toğaçar et al.^25^	CNN	It is able to distinguish between normal and aberrant pictures	Medical images and other fields cannot use it
Rehman et al.^26^	CNN	It offers a higher success rate	Not mentioned
Badža et al.^27^	CNN	To use a convolutional neural network to categorize various kinds of brain tumors	Not mentioned
Narmatha et al.^28^	fuzzy brain-storm optimization (FBSO)	It is powerful and highly successful	If only the image's entropy is higher, a clear image will be displayed
Noreen et al.^22^	Inception-v3 and DensNet201	Using this strategy improves the model's capacity to classify tumors	The pre-trained models require fewer layers to be trained
Mohammad et al.^29^	Blockchain-based CNN	Secure CNNs with added blockchain layers for extracting features	Not mentioned
		Robust under different types of attacks with consistent and accurate performance	

To sum up, several recent studies applied deep learning to the classifying of brain tumors, and they achieved good results (Phaye et al.^16^; Pashaei et al.^17^; Gumaei et al.^18^; Haq et al.^21^; Noreen et al.^22^; Hashemzehi et al.^23^; Özyurt et al.^24^; Toğaçar et al.^25^; Rehman et al.^26^; Badža et al.^27^; Mohammad et al.^29^). However, a key challenge remains in existing methods: proper tumor segmentation, which is usually performed as a manual process or by less precise algorithms. It can be instrumental in the final decision-making process. In this regard, the innovative FFO-based segmentation step is our solution to this problem. FFO offers a very profitable approach to segmentation which increases the accuracy of the segmentation of the brain tissue compared to the traditional methods. In a nutshell, this result in addition to providing the foundation for the subsequent parallel CNN architecture to extract meaningful features, can lead to higher tumor classification precision.

## Research methodology

In this study, first, the specifications of the dataset used in the current research for classifying brain tumors in MRI images are described. Then, the details of the proposed method’s steps for classifying brain tumors in MRI images are presented.

### Data

In the current research, BRATS2018 database samples were used. The samples of this database are placed in two classes, HGG and LGG. The HGG class consists of high-grade malignant tumor samples obtained from three data sets named "2013" (20 samples), "CBICA" (88 samples), and "TCIA" (102 samples). Conversely, the LGG category comprises samples with malignancies of low-grade. This class has 65 samples obtained from the TCIA dataset. Each BRATS2018 database sample is described using four modalities: T1, T1GD, T2, and T2 FLAIR. All of these images have dimensions of 155 × 240 × 240 voxels, with the volume of each voxel set to 1 cubic millimeter. In the process of evaluating the proposed method, an axial slice containing the tumor region is extracted from each database image. Such samples may have several masses at varying levels, and in such cases, more than one axial slice of the MRI picture is recovered. 500 2D pictures are extracted from the BRATS2018 samples as a consequence of this method. Out of the total samples, 255 are classified as HGG and 245 are classified as LGG. All images were normalized using min–max scaling between 0 and 1 to ensure consistent pixel intensity across the dataset. Also, to improve contrast and reduce noise, histogram equalization was applied to each image. Since all samples of BRATS2018 only include the brain tissue, therefore no skull stripping was performed on samples of the dataset. Finally, images were resized to a dimension of 200 × 200 pixels using bilinear interpolation to ensure compatibility with the CNN architecture.

### Proposed method

**Figure 1:**
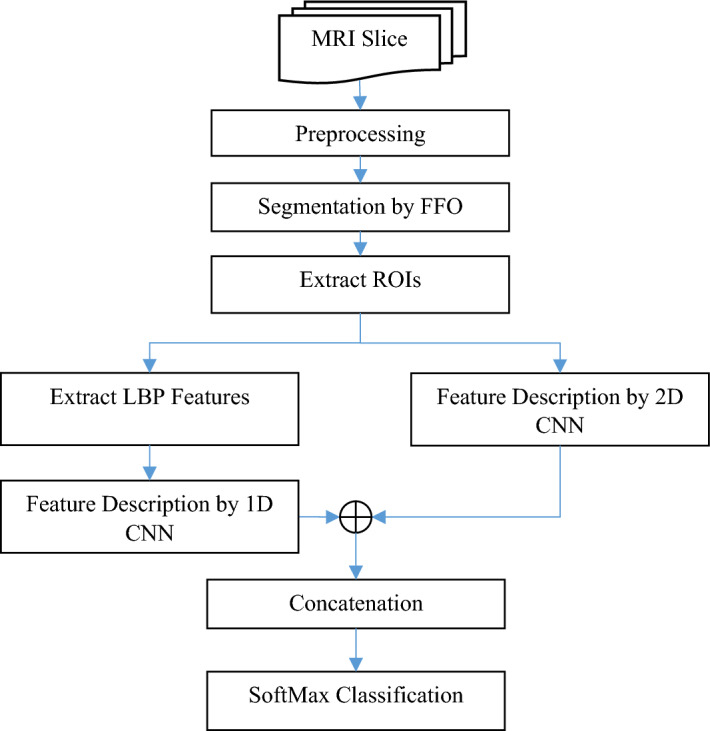
Block diagram of the proposed method.

This paper introduces a new model for processing MRI images and brain tumor classification. It combines optimization, deep learning, and image processing techniques, and briefly includes the following steps Fig. 1:

1. Pre-processing images
2. Segmentation of image regions
3. Classification

The pre-processing step is applied to each MRI image slice and during it, brain tissue regions are identified in the image slice and the result is used as input to other steps of the proposed method. In the second step, the FFO algorithm is used to segment MRI images. The objective of this study is to simplify the detection issue by restricting the range of pixel colors in each section of the input picture and estimating the area of the lesion in the image. Following the process of picture segmentation, a method based on thresholding is used to accurately identify regions of interest (ROIs). The extracted regions form the input of the third step of the proposed method, during which the existence of the tumor and its type in ROI is determined by a parallel convolutional neural network (PCNN) model. This PCNN model consists of two 1D and 2D CNNs in which the 2D CNN model is used for gray image processing of ROI and the 1D model to detect mass type through local binary patterns (LBP) extracted from ROI. Each of these CNN models defines the features related to the mass type through its last fully connected layer, and then the feature vectors extracted from these two models are combined based on a concatenation layer to finally recognize the mass type based on the integration of the features of two CNN models and using a SoftMax classifier.

#### Preprocessing

The first step in preprocessing database images is to remove the background of MRI images. The purpose of this operation is to remove redundant information from the image and to eliminate data that may interfere with the detection process. To remove the background from the images, the input image is first converted to binary mode using the experimental threshold of 0.05. Thus, each image pixel with an intensity less than 0.05 × 255 is replaced by 0, and each pixel with a higher intensity than this threshold is replaced by 1. Thus, a binary picture, denoted as B, is acquired. The objective is to estimate the greatest contiguous area of brain tissue in picture B that has a value of 1. The erosion operator is used to enhance the front edges of the image. The erosion operator between two binary sets A and B can be displayed as a selection of the set {z|(B_z_ ⊆ A}. In other words, the goal is to select a set of positions called z that overlaps only with the foreground segment A. After selecting the set z, the corresponding points in A are replaced by neighboring regions in B. If the erosion operator is shown with the symbol ·, then for the foreground sets A(x,y) and B(x,y) Eq. (1) is true^30^.

1 $$ \left( {A \cdot B} \right)\left( {x, y} \right) = min \{ A\left( {x + x{\prime} , y + y{\prime} } \right) - B\left( {x{\prime} , y{\prime} } \right) | \left( {x{\prime} , y{\prime} } \right) \in DB\} $$

where D_B_ is the domain of background values B. It should be noted that the background segment of MRI images is flat and uniform. In this case, B(x,y) = 0 and Eq. (1) can be rewritten as follows^30^:

2 $$ \left( {A \cdot B} \right)\left( {x, y} \right) = min \{ A\left( {x + x{\prime} , y + y{\prime} } \right) | \left( {x{\prime} , y{\prime} } \right) \in DB\} $$

The resulting region may not be contiguous and may contain holes with zero values. Given the contiguous nature of the foreground segment in MRI images, any holes within the selected region are filled with a value of 1, and all points that are not part of the selected region are assigned a value of 0. Therefore, the binary image ${B}{\prime}$ is obtained. Finally, to obtain the foreground image Eq. (3) is used.

3 $$ FG = B{\prime} \cdot I $$

where, by multiplying each pixel of the image I by corresponding bits in image ${B}{\prime}$, the background segment of the image is removed.

#### Segmentation and identification of the target region

In the second step of the proposed method, each image slice is segmented using a new approach. In the proposed method, the combination of K-Means and FFO algorithms is used to segment images. The objective of this stage is to simplify the problem by partitioning the brain area into its individual tissues. Through this procedure, it is feasible to segregate the mass area in the picture as a distinct cluster from other regions. The choice of FFO for segmentation on MRIs, offers several advantages over other optimization algorithms:

- Strong Exploration Capabilities: FFO is an innovative approach that is capable of a thorough search in the problem space and locating the best segmentation parameters. This results in correct brain segmentation of the tissue as compared to the other methods that may get trapped in local minimums.
- Balancing Exploration and Exploitation: FFO exhibits this balance through two methods: looking for new opportunities to divide the population and exploiting potential areas for reproduction. This guarantees continued exploration of diverse areas of the search space.
- Fewer Control Parameters: The FFO optimization algorithm does not need as many user-defined parameters as several other algorithms. It is particularly simple and easy to adjust to different datasets.

The above-mentioned advantages of FFO are thus especially significant for MRI image segmentation process where correct dissection of brain tissues is crucial for further tumor classification. Also, the comparisons conducted in^31^ and^32^ demonstrate the superiority of FFO over other optimization algorithms for solving various problems.

Brain images may be segmented by evaluating variations in brightness intensity to distinguish distinct areas and tissues. Thus, through optimal adjustment of threshold values for each region, it is possible to determine the correct separation of regions in the images. The proposed technique models this step in the form of an optimization problem and uses FFO to solve it. The proposed method uses an approach similar to the basic K-Means algorithm for image segmentation; however, the center of each region in the image is considered as an optimization variable and its optimal value is determined using FFO. Additionally, to expedite the convergence of the proposed segmentation algorithm, the initial FFO population is established using the K-Means algorithm. In this section, firstly, the formulation of the segmentation process in the form of an optimization problem is discussed, and then the method of solving it using FFO is explained.

Consider an image like X, which should be decomposed into K non-overlapping segments. In each MRI slice, the distinction between the two tissues is only possible by examining the spatial information and brightness intensity of the pixels of the tissue. As a result, a segment in image X can be described as a set of adjacent pixels that together have a lower intensity difference than other adjacent regions (with a common border). On the other hand, two unconnected regions A and B with similar intensity values can be considered as two separate segments. This definition specifies that the identification of each segment necessitates the examination of both the spatial information and the brightness intensity of the pixels. In this study, the optimization issue involves considering an optimization variable for each target area in the picture. This variable sets the brightness intensity threshold associated with that region. Thus, each solution vector in the proposed algorithm is encoded as a numerical vector with a length of K, each value in this vector has search limits (0.255) and specifies the brightness intensity threshold of corresponding region pixels.

In the proposed method, the membership of each pixel in a region is determined by calculating the difference of its brightness intensity with the thresholds determined in the solution vector. Thus, each pixel of the image belongs to a region with the lowest absolute value difference with the corresponding threshold in the solution vector. Consequently, the optimization process produces a solution vector that may be used to develop a potential segmentation for the given input picture. The primary goal of the optimization procedure is to ascertain the optimal segmentation of the candidate solution generated. For this purpose, it should be possible to determine the superiority of a segmentation state over other states by using the fitness evaluation function. The proposed optimization model employs a combination of three distinct criteria to ascertain the optimal segmentation state for each image:

- intra-cluster distance
- inter-cluster distance
- entropy

In order to evaluate the suitability of each solution vector based on the above criteria in the proposed method, first image X is decomposed into K regions based on solution vector S. In this process, the absolute difference between each pixel and each of the K thresholds defined in S is calculated. Then, the pixel is assigned to the region with the smallest difference relative to its corresponding threshold. By applying this operator to all the pixels, the X image is divided into K regions to obtain the Y matrix. The segmentation accounts solely for the brightness intensity of the image X and disregards the spatial information of the regions. Therefore, in the following, the obtained regions are separated based on the spatial information. In this case, the image matrix Y is segmented into its constituent contiguous regions. Subsequently, if the area of a contiguous region exceeds 0.05 of the brain region (identified during the pre-processing phase), a new unique identifier is allocated to that region, designating it as a distinct segment. After performing this process for all regions, the candidate segmentation image is decomposed based on the solution vector S into $L\ge K$ non-overlapping regions, which is used to evaluate the quality of this segmentation using entropy measures and intra-cluster and inter-cluster distances. In the proposed segmentation algorithm, the segmentation quality obtained from each solution vector is calculated using Eq. (4):

4 $$Fitness\left(Y\right)=\frac{{D}_{w}+{E}_{y}}{\alpha +{D}_{b}}$$

where $\alpha >0$ is a parameter for adjusting the effect of distance measure and it is considered as 1. The parameter ${D}_{w}$ denotes the intra-cluster distance and represents the average absolute difference in brightness intensity between the pixels of each segment with a unique identifier and its center in image Y. This measure can be formulated as follows:

5 $${D}_{w}=\frac{1}{\left|C\right|} \sum_{i=1}^{\left|C\right|}\frac{1}{{N}_{i}}\sum_{j=1}^{{N}_{i}}{D}_{{q}_{j}. {C}_{i}}$$

where C represents the set of unique segments Y and ${N}_{i}$ describes the number of pixels of the segment i.

Also, ${D}_{{q}_{j}. {C}_{i}}$ shows the brightness intensity difference of the pixel j from the center of the segment i. additionally, in Eq. (4), the parameter ${D}_{b}$ represents the inter-cluster distance, which reflects the minimum brightness intensity difference between the center of one segment and the centers of other segments in the image. in other words, to calculate the inter-cluster distance, the brightness intensity difference between the center of a segment, such as segment i, and the centers of other segments with distinct identifiers are computed. Then, the smallest of these differences is taken as the measure for this criterion. This measure can be calculated as follows:

6 $${D}_{b}=\frac{1}{\left|C\right|}\sum_{i=1}^{|C|}\underset{\forall j\in C}{\text{min}}{D}_{{C}_{i},{C}_{j}}$$

Eventually, ${E}_{y}$ in Eq. (4), represents the average entropy of the unique segments of the image Y. For this purpose, the entropy of each segment is calculated separately:

7 $${E}_{y}=\frac{1}{\left|C\right|}\sum_{i=1}^{|C|}\left(-\sum_{x\epsilon {C}_{i}}p\left(x\right)\text{log}\left(p\left(x\right)\right)\right)$$

where $p\left(x\right)$ indicates the probability of the brightness intensity of x in the segment. The proposed segmentation algorithm aims to form a state of image segmentation that maximizes the inter-cluster distance while minimizing the entropy and intra-cluster distance of each segment. By minimizing the entropy and intra-cluster distance, segments may be formed with optimal pattern uniformity. By maximizing the intra-cluster distance, it is feasible to attain a state of segmentation where the differences between both areas are maximized. The suggested technique employs Fast Fourier Transform (FFT) to identify a segmentation that effectively minimizes Eq. (4).It should be noted that high convergence speed is very important in a segmentation algorithm based on optimization techniques. Meanwhile, in addition to the search technique used, the way the initial population is formed is important. In the proposed method, the K-Means algorithm is used to determine a segment of the population, so that, approximations of the adjacent points of the global optimum can be provided for the optimization algorithm. Since adjusting the entire initial population based on the K-Means algorithm can increase the risk of the algorithm getting trapped in the local optimum; therefore, in the proposed method, ¼ of the initial population in FFO is determined based on k-means, and other solution vectors in the initial population are assigned randomly. In this case, to determine each K-Means initial solution vector, the image X is converted into a vector form and the resulting vector is divided into K clusters. Then the centers of the resulting clusters are considered as a solution vector. The steps of image segmentation by the FFO algorithm are as follows:

**Algorithm 1:**
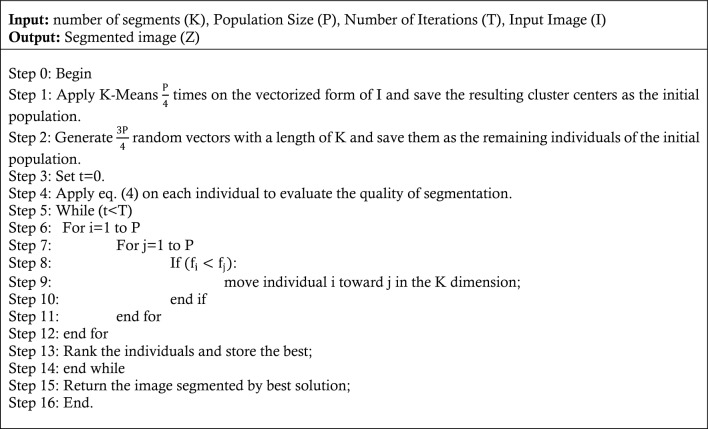
Medical Image Segmentation by FFO.

**Figure 2:**
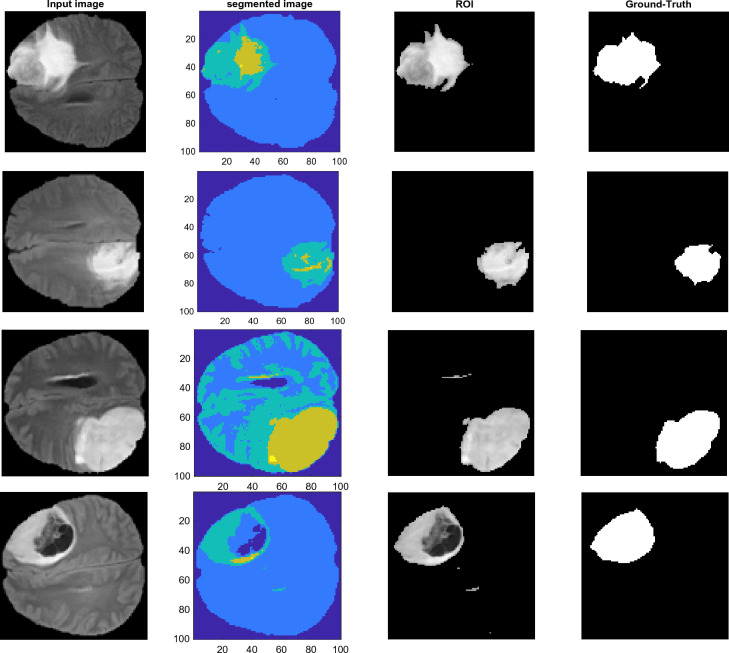
Pre-processing, segmentation, and ROI region extraction steps for some samples of the BRATS2018 database (ground-truth segmentation presented in the last column).

The segmented image obtained from the above steps is used to identify the target region. In the proposed method, the inherent features of brain tumors in MRI images are used to identify suspected tumor regions. Brain tumors in MRI images often appear as continuous regions with higher intensity (at least at their edges) compared to other regions of the brain. Therefore, in the proposed method, ROIs are determined based on the features of average brightness intensity and area. The collection of areas chosen in this phase serves as the input for the third stage of the suggested methodology. Figure 2 presents the performance outcomes of preprocessing, segmentation, and target range identification on many samples from the BRATS2018 database. Figure 2 delineates each row as it pertains to the successive steps involved in processing an image sample. Furthermore, each column showcases one of the fundamental processing phases of the suggested technique. The first column specifies the result of pre-processing, during which the redundant regions of the image are identified and removed. The second column shows the segmentation result of each image by FFO. In the third column, the ROI region extracted from the input image is given. In the fourth column, the ground-truth segmentation image is illustrated which shows the actual tumor as a bright region. In Fig. 2, the two images displayed in the first and second rows belong to the HGG class and the other two images belong to the LGG class. As shown in Fig. 2, the proposed method can identify the brain region in the input images during the preprocessing step. In addition, the segmentation step of the proposed method has an acceptable performance and can detect different brain regions with high accuracy. This proper performance in segmentation has resulted in the accurate detection of the target region in the input images, which can be seen in Fig. 2.

#### Classification

**Figure 3:**
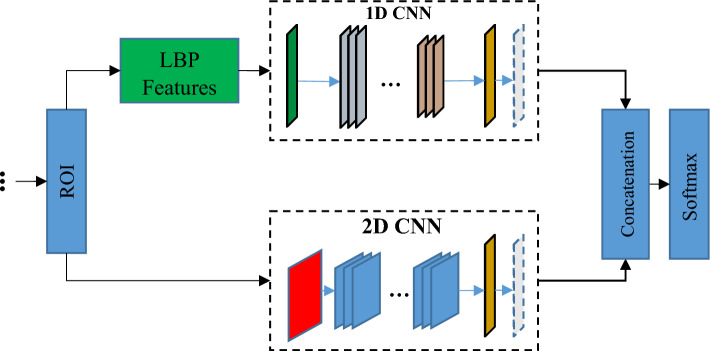
Architecture of the proposed parallel CNN model.

In the third step of the proposed method, a parallel model of convolutional neural networks (CNNs) is used to classify each ROI. The suggested parallel Convolutional Neural Network (CNN) model has two CNN components that collaborate in the task of identifying the specific kind of lesion. Figure 3 illustrates this structure. Based on the above diagram, the suggested model comprises of two components: a 1D Convolutional Neural Network (CNN) and a 2D CNN. The CNN model attempts to perform pattern learning based on local binary patterns extracted from ROI; while the 2D CNN model performs this operation based on the gray ROI matrix.

According to Fig. 3, the output of each CNN model is achieved through the weighted vector of the last fully connected layer of that model. The feature vectors of two CNN models are joined together to combine the decision results of the two models using a SoftMax classifier and create the final output of the system. The first CNN model in the proposed method is fed by LBP features, while the second CNN accepts ROI images with gray color schemes. In the subsequent sections, the classification steps for each sample utilizing this model are detailed.

##### LBP feature extraction

The LBP operator generates a binary number for each pixel according to the label of neighboring pixels in radius R. Labels are obtained by thresholding the value of neighboring pixels with a central pixel value. In this way, pixels with a value greater than or equal to the value of the central pixel are labeled 1, and pixels with values smaller than the value of the central pixel are labeled 0. Then these labels are placed together rotationally and form an L-bit number. After labeling the image by the LBP operator, a histogram of labels is defined as follows^33^:

8 $$LB{P}_{P,R}\left({x}_{c},{y}_{c}\right)= \sum_{n=0}^{P-1}s\left({g}_{n}- {g}_{c}\right){2}^{n}$$

where n is the number of labels generated by the LBP operator and the function s is defined as follows.

9 $$s\left(x\right)= \left\{\begin{array}{c}1 :x\ge 0\\ 0 :x<0\end{array}\right.$$

Uniform patterns refer to patterns that include a maximum of two transitions between 0 and 1, or vice versa, when the bits are rotated. By saving consistent patterns, a novel operator is generated, including a total of 59 distinct patterns inside the 8-pixel region. The suggested approach utilizes an adjusted model that relies on methodology^34^ to extract LBP features. In the proposed method, the segmented image is divided into N non-overlapping cells, and the LBP features of each image cell are extracted based on^34^. Then, the LBP features of each cell are described as a histogram vector of length B. As a result, each image can be represented as a vector with a length of $N\times B$. It should be noted that in the proposed method, the LBP features of each image are extracted using two radius values, R = 1, and R = 2, and the number of neighboring pixels is set to 8. As a result, the resulting feature vectors to describe the LBP features of each segmented image have a length of $2\times N\times B$.

##### Training parallel CNN models

**Figure 4:**
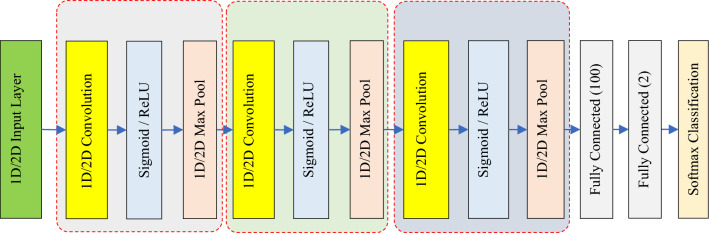
The general structure of CNN models used in the proposed classification model.

The CNN models used in the proposed method include input layers, convolution blocks for feature extraction, and necessary layers for feature classification. The general structure of these CNN models is depicted in Fig. 4.

According to Fig. 4, both CNN models used in the proposed method include 3 convolution blocks. In the first CNN model, 1D convolution and pooling layers are used to process the selected features, while the second CNN model uses 2D pooling and convolution layers. Consequently, it is inherent that the input layer of the 1D CNN model is one-dimensional, whereas the input layer of the 2D CNN model is in matrix form. The 1D CNN model utilizes the sigmoid function as its activation function, while the activation function of the 2D CNN model is uniformly set to the ReLU type in all convolution blocks. Both CNN models end with the layers necessary to classify the samples. In this approach, the dimensions of the extracted feature maps are first reduced by two consecutive fully connected layers with dimensions of 100 and 2, respectively. Subsequently, the classification of the samples into the target classes is performed using a SoftMax layer. It should be noted that in the proposed PCNN model, the output values of the last fully connected layer are combined and at the end, a classification layer is used to determine the output class of each sample. Table 2 details the configuration of the layers in the 1D CNN and 2D CNN models.

**Table 2:** Detailed configuration of layers in the proposed 1D CNN and 2D CNN models.

Layer	$1D CNN$	$2D CNN$
Input	F	F × 64
Convolution1	1D (8,16)	2D (7 × 7, 6)
Activation1	Sigmoid	ReLU
Pooling1	1D Max pooling (2)	2D Max pooling (2 × 2)
Convolution2	1D (5, 24)	2D (5 × 5, 18)
Activation2	Sigmoid	ReLU
Pooling2	1D Max pooling (2)	2D Max pooling (2 × 2)
Convolution3	1D (3, 30)	2D (3 × 3, 20)
Activation3	Sigmoid	ReLU
Pooling3	1D Max pooling (2)	2D Max pooling (2 × 2)
FC1	100	100
FC2	2	2

The configuration of each of the proposed CNN models and the determination of the number of their convolution blocks were done based on the problem conditions. Experimental tests showed that using more than 3 convolution blocks in both models leads to overfitting of the models; while the use of only one or two convolution blocks to extract features in these CNN models leads to a decrease in accuracy. The 1D CNN model is fed through a numerical vector with length F (the number of LBP features). In contrast, the 2D CNN model accepts the ROI image matrix. The CNN models are trained and used to forecast the target variable for each test sample. The output values of the final fully connected layer in each model are concatenated using a concatenation layer to produce the output of the PCNN system.

The proposed parallel CNN architecture utilizes the Adam optimizer^35^ for the gradient descent purposes during the training. An initial learning rate of 0.005 is chosen for the model, which will help to make the adjustments as precise as possible during optimization. In order to have a tradeoff between computational efficiency and model convergence, the batch size is set at 32. These hyperparameters were selected based on the empirical testing to obtain maximal accuracy and the training dataset. The training run was for 100 epochs. Additionally, the following settings were employed:

- Momentum: $${\beta }_{1}=0.9$$,$${\beta }_{2}=0.999$$
- Learning Rate Scheduler: Exponential Decay with the factor of 0.1
- Gradient Clipping: L2norm with a soft threshold
- L2 Regularization: Weight decay factor of 0.0001
- Decay rate of gradient moving average: 0.9
- Denominator offset: $${10}^{-8}$$

In the above list, factor 0.1 for learning rate scheduler declines learning rate by 10% after each training. Also, L2 Regularization helps prevent overfitting by penalizing the large weights and gradient clipping implements a soft threshold on the magnitudes of gradient updates for stability. Finally, using the denominator offset, solver adds the offset ${10}^{-8}$ to the denominator in the network parameter updates to avoid division by zero.

## Implementation results

MATLAB 2020a software is used to implement the proposed technique. In addition to applying the cross-validation methodology with ten iterations for the experimental setting, we tested the suggested method based on the predefined criteria.

### Metrics for performance evaluation

In the following, we will examine the criteria of Precision, Recall, F-Measure, Accuracy, Matthews correlation coefficient (MCC), Critical success index (CSI). In this paper, we considered two categories of targets: LGG and HGG. LGG was considered as negative category and HGG as positive category. After classification of samples, each test sample may fall in the one of the following 4 groups:

- TP: The number of samples belonging to the HGG category and correctly identified.
- TN: The number of samples belonging to the LGG category and correctly identified.
- FP: The number of samples that were in the LGG category and were mistakenly placed in the HGG category.
- FN: The number of samples that were in the HGG category and were mistakenly placed in the LGG category.

The quality of a measured value's agreement with an actual or standard value is called accuracy. Stated differently, the quantifiable accuracy of the tool lies in its capacity to quantify precise values.

10 $$Accuracy= \frac{TP+TN}{TP+FP+TN+FN}$$

By aggregating the true and false instances, genuine situations may be classified into several categories. Recall is obtained by dividing the number of true items by the total number of items in a given class. The F-measure's weighting value may be calculated using accuracy and recall data. When assessing classification quality and characterizing the weighted average of precision and recall, the F-measure is a useful metric. This measure's value ranges from 0 to 1, where 0 represents the worst situation and 1 represents the ideal one. This parameter was computed using Eq. (13) as follows:

11 $$Precision=\frac{TP}{TP+FP}$$

12 $$Recall=\frac{TP}{FN+TP}$$

13 $$F-Measure=\frac{2*Precision*recall}{Precision+recall}$$

The accuracy of binary classifications is evaluated using the correlation-based Matthews Correlation Coefficient, which has a range of − 1 to + 1 and accounts for true positives, false negatives, and class imbalance. Using, the MCC is calculated

14 $$MCC=\frac{\left(TP\times TN\right)-(FP\times FN)}{\sqrt{\left(TP+FP\right)}(TP+FN)(TN+FP)(TN+FN)}$$

With a range of 0 to 1, CSI calculates the percentage of accurately predicted positive occurrences to all events. It is sometimes referred to as the genuine talent statistic or threat score. The CSI is determined by using

15 $$CSI=\frac{TP}{(TP+FP+FN)}$$

### Comparative analysis

We evaluated the proposed approach in four different ways, which we will discuss below. In addition, we compared the proposed method to Rammurthy et al.^20^ and DACBT^21^ literature.

Proposed method: The proposed approach combines deep learning, image processing, and optimization techniques.

Prop.(without FFO): This is a way of not doing segmentation of images and instead feeding the entire image to convolutional neural network (CNN) models. In this manner, the different regions of the image texture are supplied to the CNN entirely and without segmentation, allowing the model to deliver the necessary diagnostic.

Prop.(1DCNN): To identify the target range, we first used the segmentation operation. We then feed only the characteristics relevant to the target range to a 1D convolutional neural network supplied through the LBP features. This network then generates the output we need to determine the desired range.

Prop.(2DCNN): This happens when we provide a two-dimensional neural network an image of the target area, and the network detects the target area for us. The FFO algorithm's simulation parameters are displayed in Table 3.

**Table 3:** Simulation parameters for FFO.

Parameter	Value
Population size	150
Number of iteration	500
Number of segments	5

**Figure 5:**
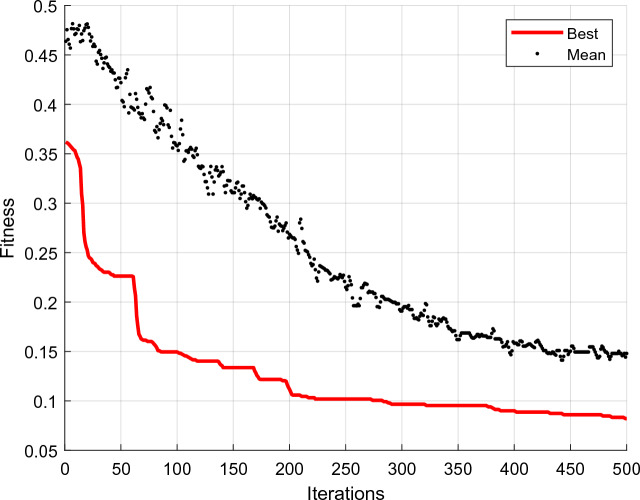
Fitness changes for segmentation of one of the database images using FFO.

Graph 5 is an example of the graph of fitness changes for the segmentation of one of the database instances. The population's average fitness is displayed in black dots in this Fig. 5, while the best fitness depicted is indicated in red.

### Experimental results

**Figure 6:**
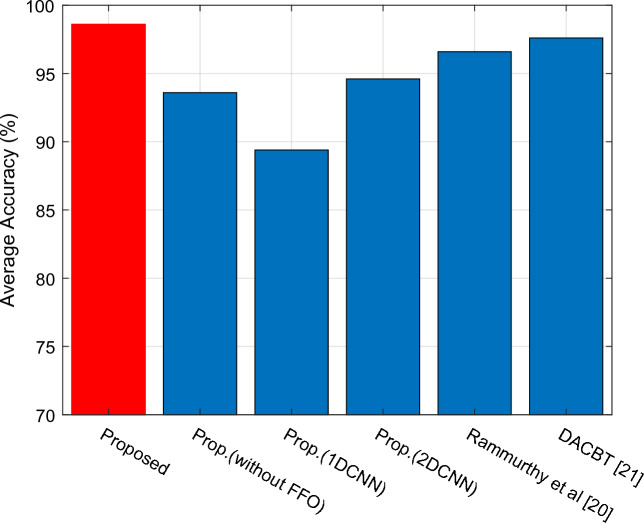
Evaluation of average accuracy.

The average accuracy of the comparison approaches and the suggested method is displayed in Fig. 6. The comparison technique DACBT^21^ has also obtained 97.60, whereas the proposed approach has reached 98.60 and Prop. (2DCNN) has reached 94.60. Furthermore, when comparing our suggested technique to Prop. (without FFO), which did not use the FFO algorithm for image segmentation, our method exhibits a 5% improvement in performance. Our proposed technique demonstrates superior performance compared to previous methods in brain tumor classification and brain MRI image processing.

**Figure 7:**
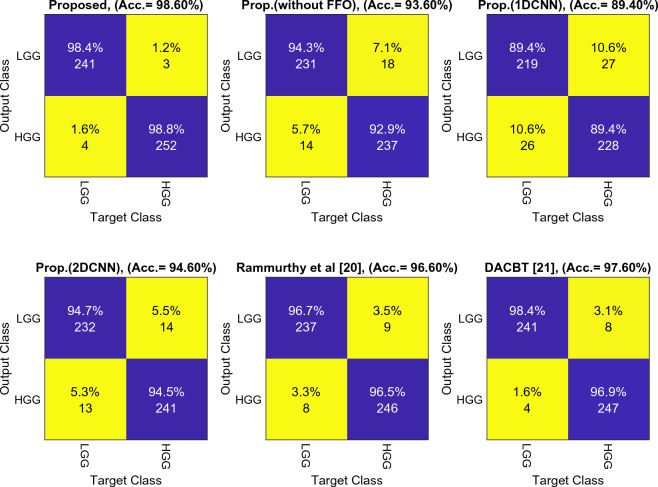
Evaluation of approaches using a confusion matrix.

The confusion matrix is depicted in Fig. 7. As can be observed, the proposed method for both target categories was able to conduct the classification more accurately and with less errors. In a database with 500 samples that we used, the number of classification mistakes with this method is equivalent to 7, which is decreased by at least two times when compared to other methods. Our classification accuracy here is higher than that of the other methods, by 1% as compared to the method DACBT^21^.

**Figure 8:**
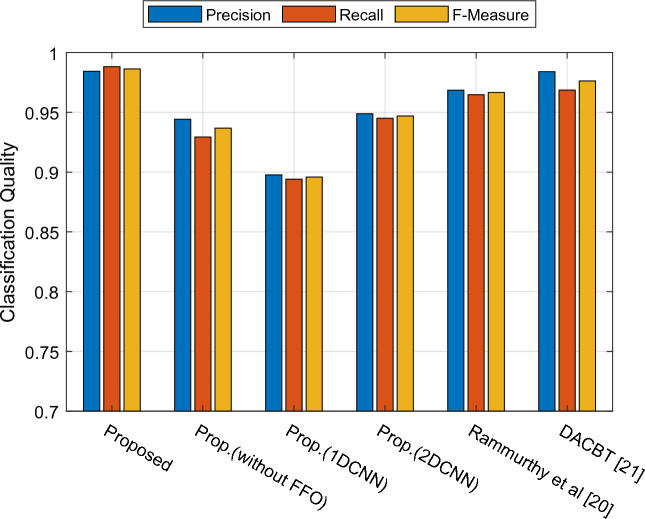
Evaluation of the classification's quality.

Figure 8 details precision, recall, and F-measure. The suggested technique achieved 0.9844 on the precision criterion, Prop. (2DCNN) achieved 0.9488, and the comparison method DACBT^21^ achieved 0.9841. In addition, the suggested technique achieved 0.9882 in the recall criterion, Prop. (2DCNN) achieved 0.9882, and the DACBT^21^ comparable method achieved 0.9686. In terms of the F-measure criterion, the suggested technique achieved 0.9863, the proposed method (2DCNN) achieved 0.9470, and the comparison method DACBT^21^ achieved 0.9763. Based on the illustrations, the suggested technique has shown exceptional accuracy in categorizing brain tumors from brain MRI data. Significant indicators have shown favorable outcomes in the diagnosis and classification of brain cancers. Such a high degree of accuracy and capacity may assist in the timely identification of brain tumors and the development of appropriate medical treatment.

**Figure 9:**
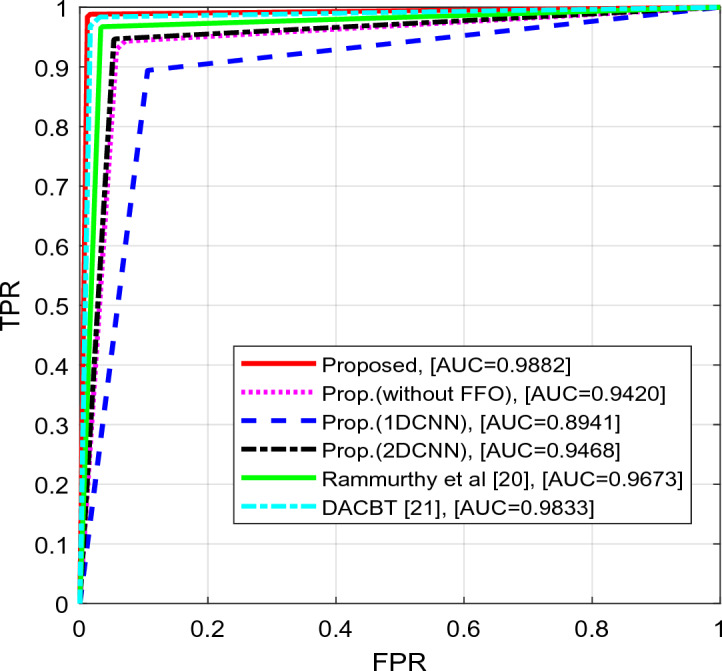
ROC curve evaluation.

Figure 9 shows the ROC curve. Based on this curve, our method has achieved a relatively high area under the ROC curve of 0.9882, while Prop.(2DCNN) has scored 0.9468 and the comparative method DACBT^21^ has scored 0.9833. An increase in the area under the ROC curve indicates a decrease in the false positive rate (FPR) and an increase in the true positive rate (TPR). These results demonstrate that the method under investigation has simultaneously delivered superior performance. It excels in reducing the FPR and enhancing the TPR, consequently significantly increasing the area under the curve compared to other methods.

**Figure 10:**
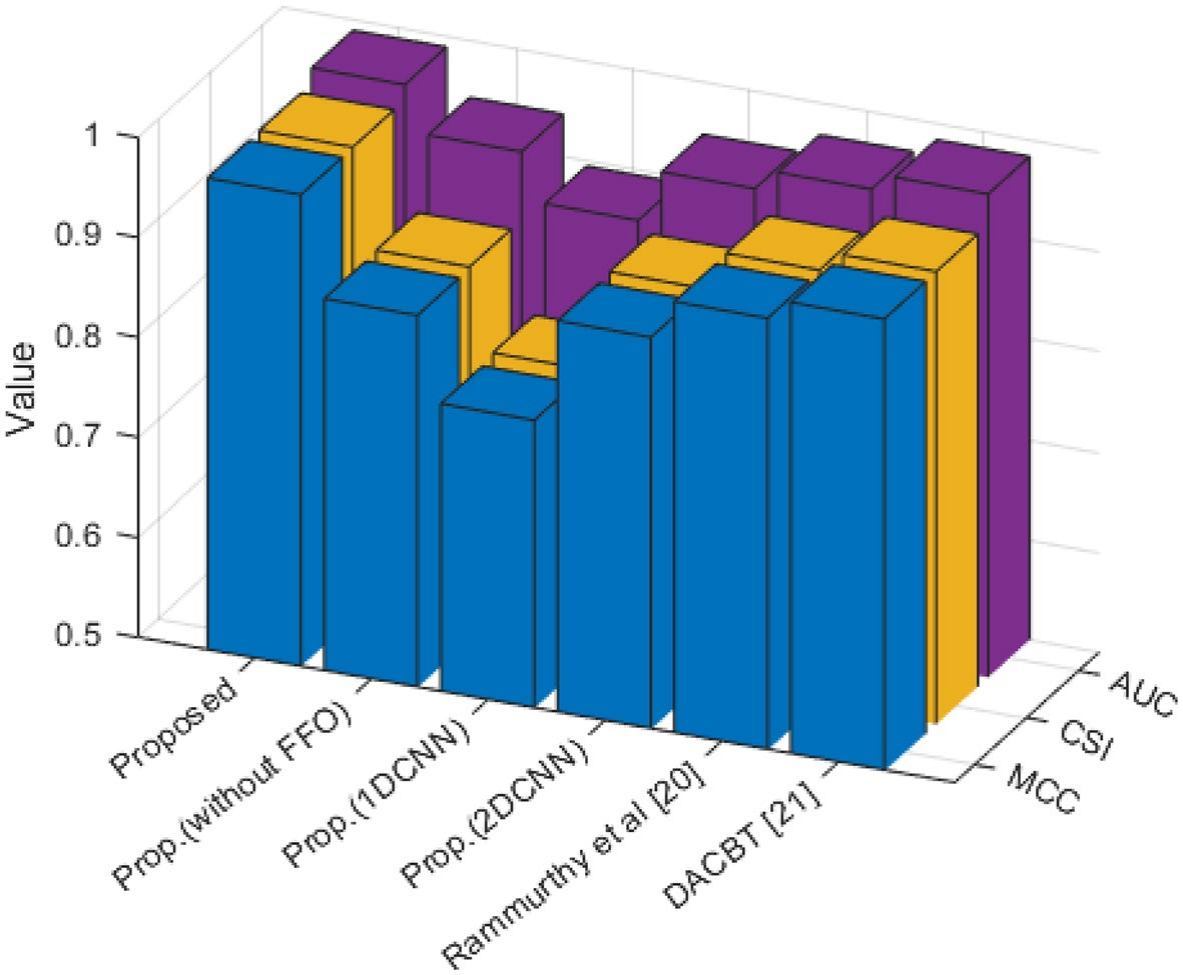
Evaluation of MCC, CSI, AUC criteria.

Figure 10 shows that our proposed method's MCC, CSI, and AUC criteria evaluations are, respectively, 0.9720, 0.9726, and 0.9882. Based on the results, our method performs 1.99%, 1.99%, and 0.49% better in terms of MCC, CSI, and AUC, respectively, than the comparative methods.

**Figure 11:**
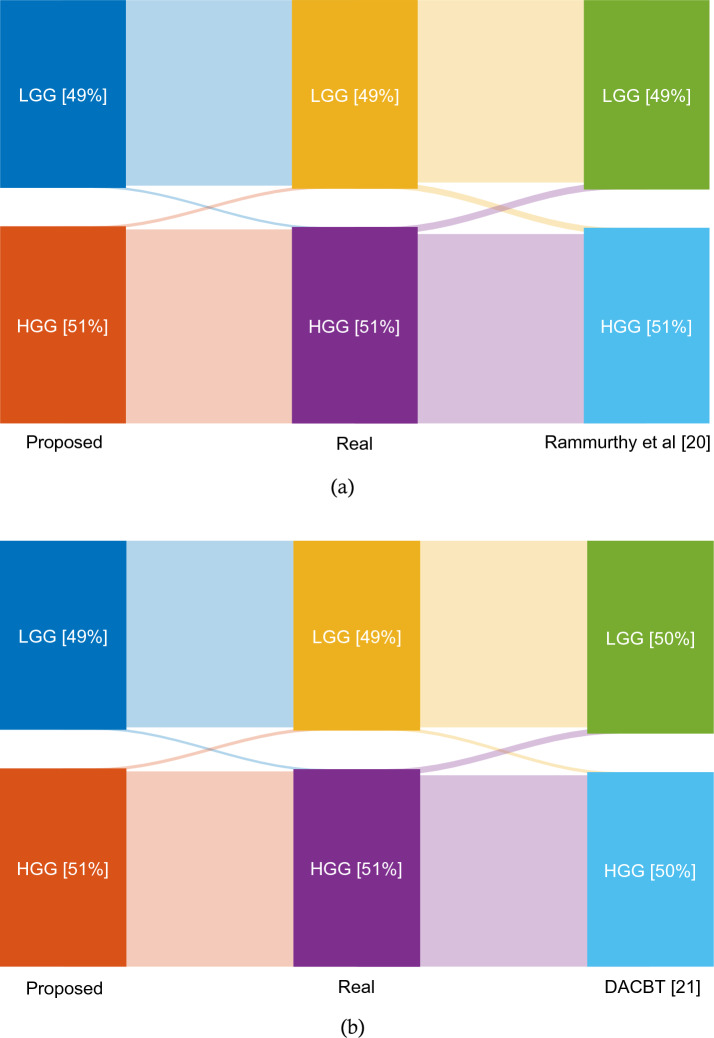
Comparing the classification quality of the proposed method with other methods based on Sankey diagram.

Figure 11 illustrates the Sankey diagram. We compare the performance of the proposed classification model in Fig. 11a with the method of Rammurthy et al.^20^. Notably, the connecting lines coming from the method we propose appear narrower than those emanating from the comparator method. This graphic representation illustrates the disparity in the output outcomes, highlighting the possible advantage and effectiveness of our suggested approach. In addition, the samples of the proposed approach, together with real samples and DACBT^21^, are shown in 11b. The proposed method results in narrower communication lines than the comparison approach. In general, our technique has less error in misclassifying samples of each class and has been able to diagnose all forms of brain tumors more accurately.

According to Table 4, our proposed method worked with an accuracy of 98.6% and a MCC of 0.9720. This performance is significantly better than the comparative approaches. For example, our proposed method outperforms the DACBT method, which has an accuracy of 97.6% and an MCC of 0.9521. It can also be seen that our proposed method outperforms the 1D and 2D CNN methods, as well as the method described by Rammurthy et al.

**Table 4:** The efficiency of the proposed method in comparison to alternative approaches.

Methods	Precision	Recall	F-Measure	Accuracy	MCC	CSI
Proposed	0.9844	0.9882	0.9863	98.6000	0.9720	0.9726
Prop.(without FFO)	0.9442	0.9294	0.9368	93.6000	0.8721	0.8736
Prop.(1DCNN)	0.8976	0.8941	0.8959	89.4000	0.7879	0.7918
Prop.(2DCNN)	0.9488	0.9451	0.9470	94.6000	0.8920	0.8939
Rammurthy et al.^20^	0.9685	0.9647	0.9666	96.6000	0.9320	0.9332
DACBT^21^	0.9841	0.9686	0.9763	97.6000	0.9521	0.9527

The obtained results show the higher level of accuracy compared to current methods (DACBT^21^, Rammurthy et al.^20^), but computational efficiency and scalability are two other crucial things that need to be considered for implementation in a real-world scenario.

The proposed parallel CNN architecture leverages two CNNs: a 1D CNN with 196,580 and a 2D CNN with 8,203,580 learnable parameters, for a total of around 8.4 million parameters. This particular parameter count may directly determine the memory needs during training and inference.

The training model was based on a NVIDIA RTX 4050i GPU. During the training process, we employed the Adam optimizer with batch size 32. The amount of memory for model parameters (running the trained model on other machines) is about 33.5 MB. This value is considered low in terms of memory, allowing the model to be used on machines with a wide range of memory capabilities compared to approaches with significantly higher memory requirements. Also, for training the model, in addition to the mentioned required memory, about 3.5 MB is required for optimizer states. Additionally, considering batch size of 32, about 16.77 MB is required for batch data. This means that the required Memory per batch in training the proposed model is about 53.8 MB which is significantly lower that compared methods.

The present training results show good capacity on the dataset of 500 images in a batch size of 32. Nevertheless, in case of much bigger datasets, the memory footprint per batch is increased as the amount of data in the batch is directly proportional to the memory footprint. This could make using training methods like gradient accumulation or mixed-precision training necessary to achieve larger batch sizes on the same hardware. Furthermore, a deep analysis with larger datasets is essential for the model's further scalability in relation to the increasing of input data. While training time increases linearly based on current observations, further investigation is needed.

The proposed model reaches high accuracy with a low number of parameters which seems to be a suitable solution that preserves both accuracy and computational efficiency. The memory footprint during the training can be controlled either using a right hardware or the training strategy. Nevertheless, for the biggest datasets, exploring such techniques as transfer learning or making use of pre-trained models for tasks similar to each other could be a great way to proceed with the research in order to reach the highest scalability.

## Conclusion

In this paper, a hybrid model made up of quick optimization algorithms and parallel convolutional neural networks is used to examine the categorization of brain cancers in MRI data. Brain tumors have been successfully classified by this model with high accuracy and low complexity. The model's efficient optimization method and use of parallel convolutional neural networks have contributed to enhancing the model's performance and extraction of features. We used the criteria of accuracy, precision, recall, and f-measure to evaluate the acquired data. Following 10 iterations, we evaluated the results using the cross validation methodology. When compared to other methods currently in use, our performance was better. Accordingly, the testing findings demonstrate that, with accuracy and precision of 1% and 0.03, our proposed approach outperformed the comparison methods.

## Data Availability

All data generated or analysed during this study are included in this published article.
